# A Novel Invention of a 3D Printed Mould for Calcaneum Replacement Post Total Calcanectomy for a Recurrent Aggressive GCT of the Calcaneum

**DOI:** 10.1055/s-0045-1809420

**Published:** 2025-07-01

**Authors:** Goh Boay Heong Eyrique, Tee Kok Keat, Aaron Gerarde Paul

**Affiliations:** 1 Orthopedic Oncology Unit, Orthopedic and Traumatology Hospital Queen Elizabeth I and II Sabah, Kuala Lumpur, Malaysia

**Keywords:** calcaneum, giant cell tumors, oncology, printing, three-dimensional, calcâneo, impressão tridimensional, oncologia, tumores de células gigantes

## Abstract

Advances in image processing have led to the clinical use of 3D printing technology, providing surgeons with realistic physical models of anatomy that enable them to recreate accurate bone structures. 3D-printed molds can play a central role in surgical replacements, offering both efficiency and cost-effectiveness. This case report describes the innovative creation of an anatomical calcaneum using a 3D printer. A patient presented with a recurrent aggressive stage of calcaneal giant cell tumor, which necessitated a complete resection, leaving a large void that required reconstruction. This paper outlines the 3D printing methodology used from pre-operative printing of the prototype, through the surgical procedure, to post-operative care. 3D images of the calcaneum were extracted from a CT scan and edited using 3D modelling software to print a hollow-shelled calcaneum. The printed prototype was created in two halves and sent for gas sterilization. After resecting the diseased calcaneum, the mold was filled with bone cement and clasped together with a proline mesh in between, for soft tissue attachment. Once the cement set, the shell was removed, and the shaped bone cement calcaneum was implanted into the patient with screws. The surrounding soft tissues and Achilles tendon were sutured to the mesh. Post-operatively, the patient was kept in a plaster dorsal slab for six weeks to allow for soft tissue incorporation. Six weeks after surgery, the patient began weight-bearing activities with an aesthetically shaped foot. This method for bone reconstruction is efficient, economical, and reproducible.

## Resumo

Avanços no processamento de imagens levaram ao uso clínico da tecnologia de impressão tridimensional (3D), dando aos cirurgiões modelos físicos anatômicos realistas para recriação precisa de estruturas ósseas. Moldes impressos em 3D podem desempenhar um papel essencial em substituições cirúrgicas, oferecendo eficiência e boa relação custo-benefício. Este relato de caso descreve a criação inovadora de um calcâneo anatômico usando uma impressora 3D. Um paciente apresentou uma recidiva de tumor de células gigantes do calcâneo em estágio agressivo, com necessidade de ressecção completa, deixando um grande espaço vazio que exigia reconstrução. Este artigo descreve a metodologia utilizada desde a impressão 3D pré-operatória do protótipo, passando pelo procedimento cirúrgico, até os cuidados pós-operatórios. As imagens 3D do calcâneo foram extraídas de uma tomografia computadorizada e editadas usando um *software* de modelagem 3D para impressão do osso como uma concha oca. O protótipo impresso foi criado em duas metades e enviado para esterilização a gás. Após a ressecção do calcâneo acometido, o molde foi preenchido com cimento ósseo e as duas metades foram unidas, com uma tela de prolene entre elas, para fixação nos tecidos moles. Após a cura do cimento ósseo, a concha foi removida e o calcâneo moldado foi implantado no paciente com parafusos. Os tecidos moles circundantes e o tendão calcâneo foram suturados à tela. No pós-operatório, o paciente utilizou uma placa dorsal de gesso por 6 semanas para permitir a incorporação dos tecidos moles. Seis semanas após a cirurgia, o paciente iniciou atividades com suporte de peso no pé esteticamente moldado. Este método de reconstrução óssea é eficiente, econômico e reprodutível.

## Introduction

Advances in image processing have led to an increasing clinical usage of 3D printing technology. The CT or MR images can be converted to 3D format files, giving a perfect anatomical model. This technology allows versatility in the design process and enables efficient production of both off-the-shelf and personalized anatomy that tailors to specificity. 1 In the field of orthopaedic oncology surgery, 3D-printed molds and instrumentation can be used to address bony restoration after a wide resection as part of tumor resection protocol. Although conventional metal implant replacements are available, costs can be exorbitant. In our case, the 3D anatomical shell is printed at minimal cost, and the bone cement used to fill it is also low in cost. This paper reviews the surgical technique and outlines the basics of 3D printing technology and its possible applications in orthopaedic surgery with its potential future impact.

## Case Report

**Fig. 1:**
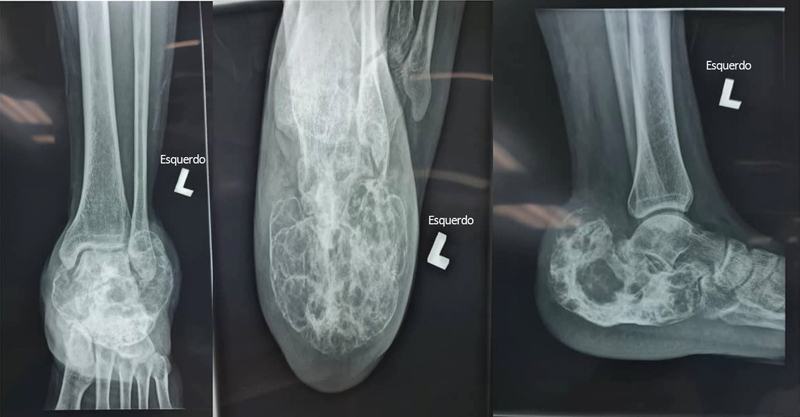
Radiograph imaging of the L pathological calcaneum in ( **A** ) AP view ( **B** ) axial ( **C** ) lateral.

**Fig. 2:**
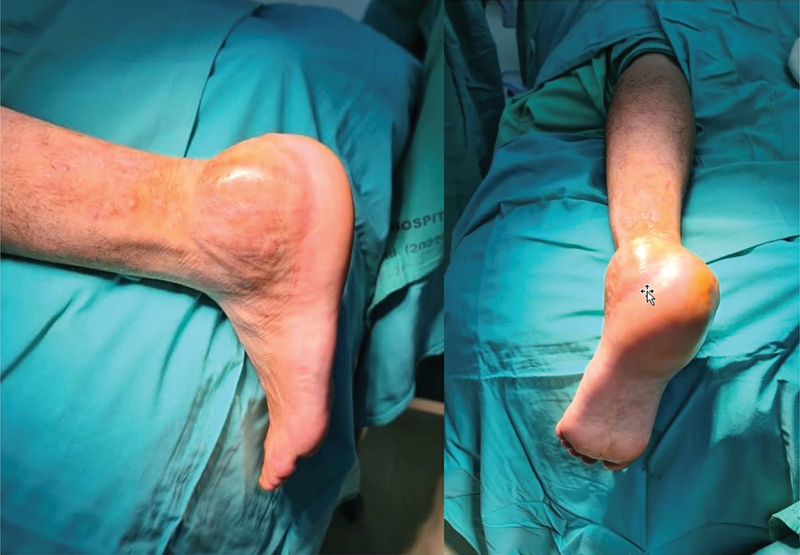
Clinical picture of the left pathological foot.

This innovative surgical treatment was performed on a patient who presented to our institution with a recurrent GCT of the calcaneum. The patient was initially treated 18 months prior with a series of Denosumab and an extended curettage. He presented with symptoms of swelling and pain over the left heel ( Figs. 1 and 2 ).

## Technique Description 3D Printing

- Preoperative planning

Various methods of 3D printing exists, however all of them share the same principles and step-wise process. 1 Although 3D printing has gained traction over the years, it is not widespread given its steep learning curve and limited knowledge. 2 As the case was an oncological pathology – it is crucial that the high standards of oncological resection principles are upheld. Patient will have to go through the same standard clinical assessment with proper imaging: A CT scan and an MRI of the foot to delineate the soft tissue extension and neurovascular involvement. Once the diagnosis is established the surgical resection margins are determined: an anticipation of a significant void as a total calcanectomy was planned. The resected calcaneum will need to be replaced or reconstructed to facilitate weight bearing. A CT of the normal contralateral calcaneum was needed for the mold creation.

- Requirements for the surgery

**Fig. 3:**
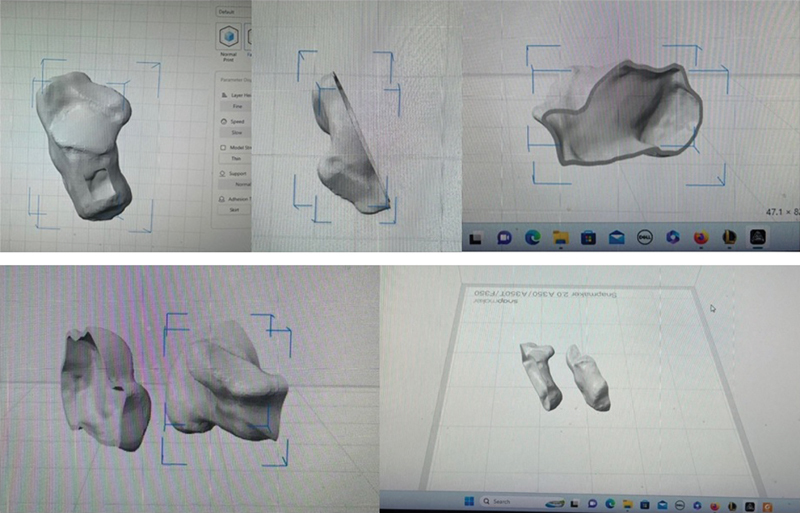
( **A** ) The creation of the 3D model from existing 3D reconstruction of the CT ( **B** ) superior view of the created halve of the calcaneum ( **C** ) the sagittal view of the created half of the calcaneum ( **D** ) creating a mirror image for printing ( **E** ) the final designs for printing in a 3D printer platform.

A standard 3D printer was required. Examples were by Kokoni Smart, Bambulab, Creality and etc. A prior CT scan and MRI was required to obtain the 3D models. These 3D models were generated from Slicer (slicer.org) and then imported into a 3D modeling software (Fusion 360) ( Fig. 3 ). The final designs can be printed using several printer-specific software. The model of the anatomical printing needs to be prepared prior to surgery and sterilized – the 3D model was sterilized with gas sterilisation as the PLA (Polylactic Acid) material used was heat labile.

- Indications and contraindications

This method of replacement follows a wide local excision of the underlying tumor avoiding the need for an amputation. Limb salvage surgery comprises surgical techniques designed to resect musculoskeletal extremity tumors and subsequently reconstruct a limb with an acceptable oncologic, functional, and cosmetic result. 3 Relative contraindications are pathologic fractures, neurovascular encasement, and a poorly placed biopsy tract. Limb salvage surgery is the preferred treatment of musculoskeletal extremity tumors in the modern era because limb salvage surgery has proven not to compromise survival or recurrence when compared with amputation. 3

- Surgical anatomy

**Fig. 4:**
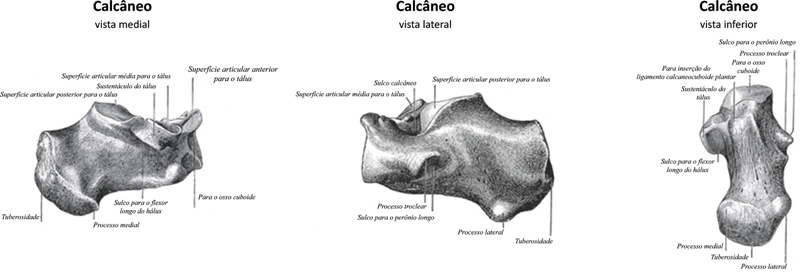
Anatomy of calcaneum ( **A** ) medial ( **B** ) lateral ( **C** ) inferior. Source: Luijkx T, Elthokapy M, Gregory L, et al. Calcaneus. Reference article, Radiopaedia.org.

The calcaneus is an irregular, roughly cuboidal bone situated below the talus forming the core of the heel ( Fig. 4 ). 4 The posterior part of the calcaneus is circular, with three facets. The superior facet is separated from the calcaneal tendon by the retrocalcaneal bursa. The middle facet provides the attachment site for the Achilles tendon. The inferior facet is continuous with calcaneal tuberosity on the plantar surface. Superiorly is a cartilage-covered facet (middle talar articular facet) for the corresponding middle facet of the head of talus as part of the subtalar joint. The anterior surface has a convex articular facet for the cuboid bone articulation.

- Surgical technique

**Fig. 5:**
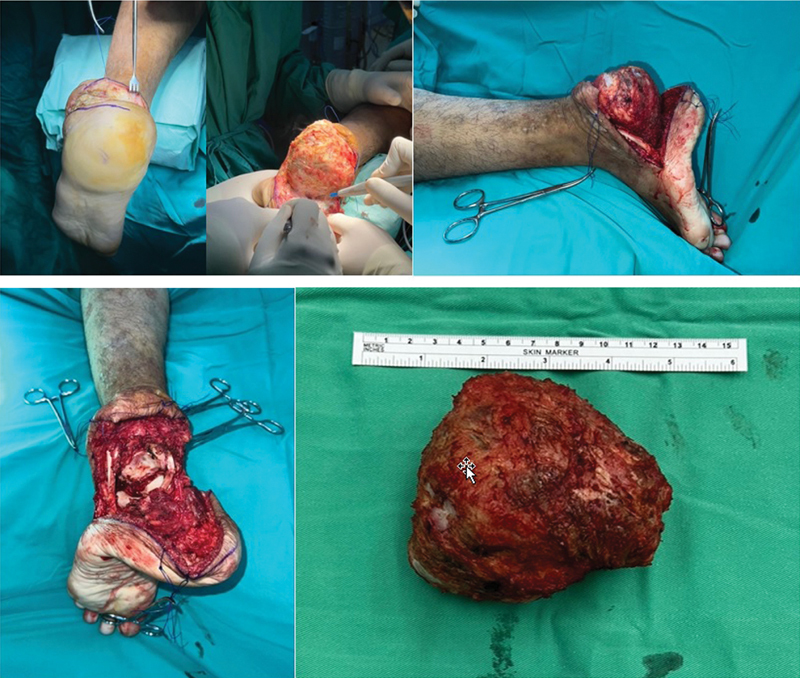
Total calcanectomy with Cincinati approach to the calcaneum ( **A** ) Cincincati approach incision over the heel ( **B** ) Wide resection of the pathological calcaneum ( **C** ) sagittal view of the pathological calcaneum ( **D** ) Voided space post total calcanectomy ( **E** ) Excised recurrent Giant Cell Tumour of the calcaneus.

All patients will adhere to strict oncological resection principle of an en bloc resection with a clear resection margin. Surgical resection is crucial in the treatment of primary solid tumors, resection at tumor margins remains a concern, inadequately resected margins facilitating tumor recurrence. 5 Post excision of the tumor – ensure no macroscopic retainment of tumor. The operation was performed in the prone position using a Cincinnati incision 6 ( Fig. 5 ). The total calcanectomy was performed, via en bloc resection ( Fig. 5 ). The triceps surae were tagged for reconstruction later.

**Fig. 6:**
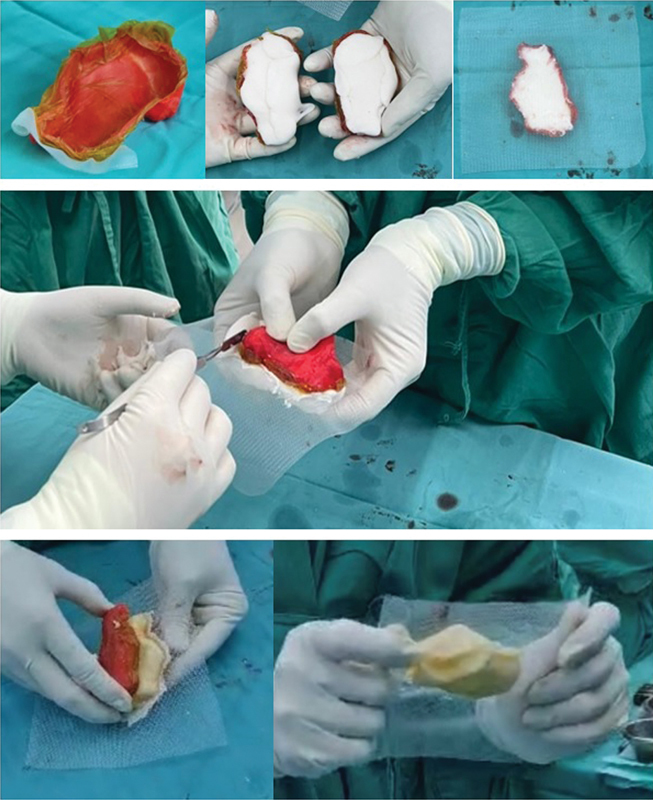
Molding of the prototype calcaneal bone cement model ( **A** ) Mold lining with ioban and liquid paraffin ( **B** ) Both molds are then filled with a packet of standard bone cement each ( **C** ) A sheath of prolene mesh is placed between before clasping both together awaiting consolidation. ( **D** ) Clasping of both molds with cement and proline mesh while the assistant clears off the extruded cement. ( **E** ) Peeling the printed mold off the calcaneal cement ( **F** ) The cement calcaneum with the proline mesh incorporated in the center for soft tissue attachment.

The sterilized printed mold is prepared for molding of the calcaneus for implantation. Each halves of the printed mold was lined with ioban and coated with liquid paraffin ( Fig. 6 ). Both the molds are then filled with one packet of standard bone cement each. The 2 molds are then clasped together with proline mesh in between. The prolene mesh serves to function as an anchor for the soft tissue reconstruction. The extruded cement upon clasping is removed while in liquid form before hardening ( Fig. 6 ).

**Fig. 7:**
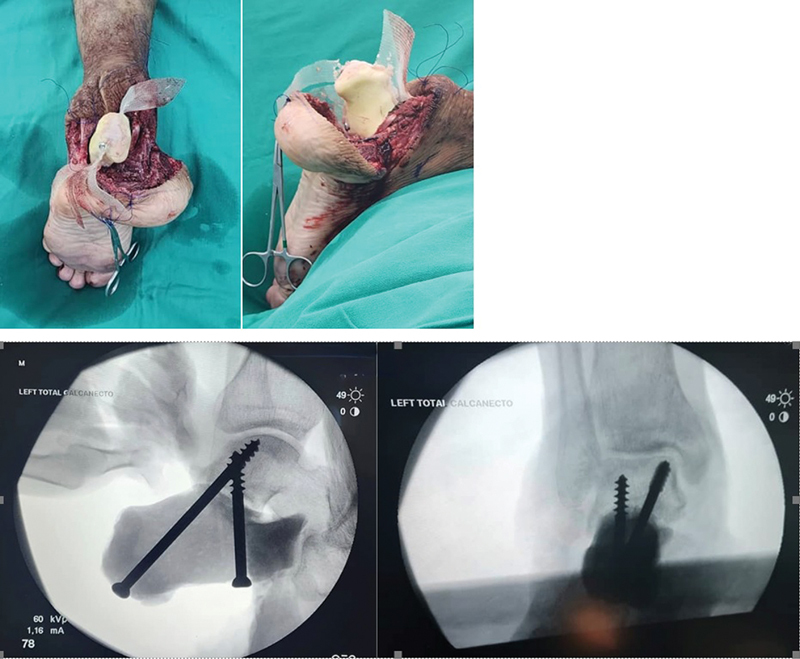
The calcaneal cement inserted into the resected calcaneal space for reconstruction. ( **A** ) Posterior view ( **B** ) lateral view Intraoperative Image Intensifier images of both inserted cancellous screws. ( **C** ) Lateral view ( **D** ) AP view.

Due to the physical properties of the polylactic acid, the hardened mold shell softens during the exothermic phase of the cement setting, enabling it to be peeled off easily ( Fig. 6 ). This molded calcaneum is then inserted to the resected calcaneal space. The Tricep surae was sutured to the posterior calcaneal aspect of the proline mesh. The proline mesh was trimmed to areas that required attachments. The Talocalcaneal joint is stabilized with the insertion of a partially treaded cancellous screws ( Fig. 7 ). Serial drill bits of increasing sizes were used to gradually dilate the tract to prevent cement from cracking.

Subsequently, after the insertion of the screws, the Tendon Achilles was sutured to the mesh over the posterior part of the calcaneum with a size 2 Ethibond. The calcaneo-cuboid joint was reconstructed with the mesh around the joint with its surrounding ligament. Alternatively – another screw from posteriorly could be inserted across the calcaneocuboid joint ( Fig. 7 ).

A drain was applied and the subcutaneous tissue was closed with Vicryl 1 and skin with Dafilon 3/0. The wound is dressed with antibiotic cream and plaster and supplemented with fluffed gauze and bandaged.

- Postoperative management

The drainage tube is kept for 3–5 days till the wound was epithelised. Sutures were removed 2 weeks post-surgery. A postoperative below-knee dorsal slab was applied for 8 weeks. Subsequently, a progressive passive and active movement of the ankle without weight for an additional 6 weeks to allow Tendon Achilles to incorporate. Partial weight-bearing with crutches was allowed at 6 weeks after surgery and full weight-bearing at 3 months. This patient will be followed up with periodic clinical and radiological examinations. Functional results were evaluated according to the system proposed by the International Society of Limb Salvage and approved by the Musculoskeletal Tumor Society. 7

- Result and Follow up

**Fig. 8:**
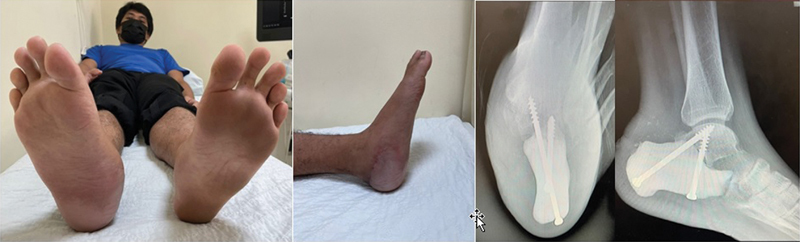
Clinical picture of ( **A** ) bilateral foot from plantar ( **B** ) lateral view of foot and radiograph of ( **C** ) axial view of calcaneum and ( **D** ) lateral foot radiograph – at 9 months follow up post surgery.

Patient was followed up post operatively in 2 weeks, 6 weeks, 3 months and 6 months after. The wound healed well without dehiscence or fat pad necrosis and patient started full ambulation after 3 months. Patient was able to walk with a fairly balanced gait and radiograph showed intact printed implant and screws ( Fig. 8 ). Patient was very satisfied with the result and rehabilitation.

- Pearls (Tip & tricks) and Pitfalls
  ○ Size and Morphology
    ▪ Anatomically snug fit without interfering surrounding structures
    ▪ Correct sizing
    ▪ Printing a 2mm thick shell is sufficient for strength and also for malleability during the exothermic cement hardening.
  ○ Molding
    ▪ Gauging the sufficient amount of cement required to fill the space without porosity within. The volume of the structure can be calculated with the 3D design software to estimate the amount of bone cement required.
    ▪ Constant pressure and precision in maintaining the clasp awaiting the cement hardening
  ○ Mechanical strength
    ▪ Implant integrity maintained to withstand load bearing
    ▪ Stable fixation across the adjacent structures
  ○ Fixation
    ▪ Incremental drilling bit to avoid cement cracking
    ▪ Use partially threaded screws without threads across the joint – to allow micro-gliding of the screws during ambulation
    ▪ Prevent too many fixations surrounding the cement causing over rigidity
    ▪ The quality, length, size, type and trajectories need to be pre-determined
    ▪ Consider additional fixations to maintain good stability
  ○ Soft tissue reconstruction
    ▪ Stable and strong fixation of the soft tissues, for example: Tendon Achilles and capsule over the calcaneo-cuboid joint.
- Complications
  ○ Infection: Both deep and superficial infection
  ○ Recurrence if margins are not clear
  ○ Skin flap necrosis
  ○ Implant fracture upon heavy weight bearing
  ○ Stiff joint leading to early onset of secondary osteoarthritis
  ○ Implant failure (screw fatigue)
- Uniqueness of the authors' technique compared with the standard technique

This method of indirect 3D printing can be reproduced and its cost is significantly less than that of direct metal 3D printing. The printing cost of the mold is minimal. The bulk of the cost is the bone cement used. This method enables an anatomical fit with near-physiological force distribution during load bearing, rather than just an odd shaped cement spacer.

## Discussion

A recurrence of a previously treated tumor, being malignant or an aggressive benign will necessitate a wider and more extensive resection – leaving behind a significant void. These voids will need to be filled to allow the distribution of forces for load bearing. The reconstruction requires a thorough 3D preoperative analysis for deformity or structural replacement, with the aim of restoring the hindfoot alignment allowing eventual ambulation via full load bearing. This complex void presents significant challenges, therefore the option of utilizing a 3D printing technology for the replacement aspect of the surgery. As replacement of the resected calcaneum is secondary, the primary importance is still maintaining clear margin resection of the recurrent tumor. Medical 3D printing was first introduced for an evaluation of intraarticular calcaneal fracture in 1997 by Kacl et al. 8 Despite the lapse of many years and the cost of production did reduce, however, the total cost in printing the entire calcaneum in titanium is still a significant financial burden to this age. 9 3D printing has evolved over the years enabling pre-operative planning, pre-contouring plates, pre-shape plates, fabricating patient-specific guides etc. 9 Given the complexity of the foot and ankle – this method definitely helped in establishing a stable fixation and anatomical replacement without significant economical strain.

## Final Considerations

This innovative approach not only promises functional recovery but also emphasizes patient comfort through reduced recovery times and enhanced anatomical fit. This is an efficient, economical and reproducible method for bony reconstruction after a complete or partial bony tumor resection. The evidence from a single case may not suffice to generate a conclusion – requires a series of cases, but this case throws light on a new novelty method that can be practiced effectively & economically. As this will set a precedent for future advancements in Orthopedic oncology worldwide especially in nations with financial constrain over health care.
